# Evaluation of Apoptosis and Autophagy Inducing Potential of *Berberis aristata, Azadirachta indica*, and Their Synergistic Combinations in Parental and Resistant Human Osteosarcoma Cells

**DOI:** 10.3389/fonc.2017.00296

**Published:** 2017-12-11

**Authors:** Pracheta Sengupta, Sukanya Raman, Rajdeep Chowdhury, K. Lohitesh, Heena Saini, Sudeshna Mukherjee, Atish Paul

**Affiliations:** ^1^Department of Pharmacy, Birla Institute of Technology and Science, Pilani, India; ^2^Department of Biological Sciences, Birla Institute of Technology and Science, Pilani, India

**Keywords:** extraction techniques, human osteosarcoma cell lines, synergism, HCR cell lines, reactive oxygen species, autophagy, caspase-mediated apoptosis, high performance thin layer chromatography

## Abstract

Cancer is a multifactorial disease and hence can be effectively overcome by a multi-constituently therapeutic strategy. Medicinal plant extracts represent a perfect example of such stratagem. However, minimal studies have been done till date that portray the effect of extraction techniques on the phyto-constituent profile of plant extracts and its impact on anticancer activity. In the present study, we have evaluated the anticancer potential of methanolic extracts of *Berberis aristata* root and *Azadirachta indica* seeds prepared by various extraction techniques in human osteosarcoma (HOS) cells. Soxhlation extract of *B. aristata* (BAM-SX) and sonication extract of *A. indica* (AIM-SO) were most effective in inducing apoptosis in parental drug sensitive, as well as resistant cell type developed by repeated drug exposure. Generation of reactive oxygen species and cell cycle arrest preceded caspase-mediated apoptosis in HOS cells. Interestingly, inhibition of autophagy enhanced cell death suggesting the cytoprotective role of autophagy. Combination studies of different methanolic extracts of BAM and AIM were performed, among which, the combination of BAM-SO and AIM-SO (BAAISO) was found to show synergism (IC_50_ 10.27 µg/ml) followed by combination of BAM-MC and AIM-MC (BAAIMC) with respect to other combinations in the ratio of 1:1. BAAISO also showed synergism when it was added to cisplatin-resistant HOS cells (HCR). Chromatographic profiling of BAM-SX and AIM-SO by high performance thin layer chromatography resulted in identification of berberine (R_f_ 0.55), palmitine (R_f_ 0.50) in BAM-SX and azadirachtin A (R_f_ 0.36), azadirachtin B (R_f_ 0.56), nimbin (R_f_ 0.80), and nimbolide (R_f_ 0.43) in AIM-SO. The cytotoxic sensitivity obtained can be attributed to the above compounds. Our results highlight the importance of extraction technique and subsequent mechanism of action of multi-constituential *B. aristata* and *A. indica* against both sensitive and drug refractory HOS cells.

## Introduction

Nature has always been the mainstay of drug discovery program for human health. Around 80% of the drugs in the market for various human diseases are either natural products or nature inspired (1). Among all the human diseases, the major contribution of natural products is in the treatment of cancer (2). There are many plant-derived anticancer agents, namely vinblastine, vincristine (*Catharanthus* sp.), epipodophyllotoxins (*Podophyllum* sp.), paclitaxel (*Taxus* sp.), and camptothecin derivatives (*Camptotheca* sp.) (1, 2). In-spite of multiple drugs being available in the market, cancer is still one of leading causes of fatality worldwide due to development of chemoresistance (3). Chemoresistance is one of the major challenges in treatment of all types of cancer and is thought to be inherent in certain populations of heterogeneous tumors or it may be acquired due to repeated drug exposure (4). Osteosarcoma, a common malignant bone tumor, is no exception affecting 2.7% of Indians. Surgery along with chemotherapy (methotrexate, doxorubicin, and cisplatin) is possible treatment options for osteosarcoma (5). However, these drugs develop chemoresistance on regular use, hence, strategies need to be developed to overcome the challenge of cancer and associated resistance.

**Figure 1:**
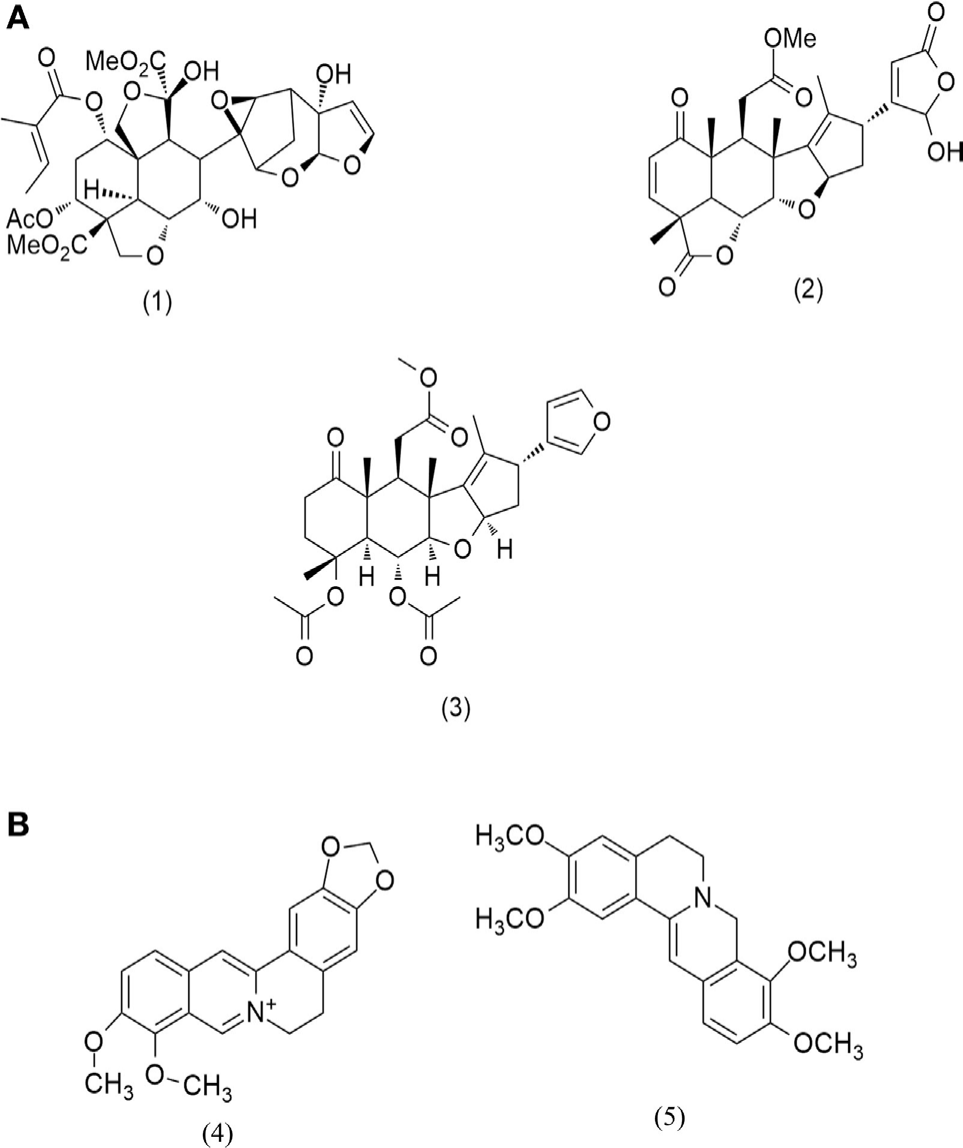
Major compounds present in **(A)** *Azadirachta indica* and **(B)** *Berberis aristata*.

In *Ayurveda*, various classical texts are found where plant crude extracts have been reported to be used in treatment of various tumors. The multicomponent system can often work in synergism, if explored judiciously. However, there are very few scientific studies conducted till date that facilitates understanding of the synergism of these herbal drugs (6). As a herbal medicine, *Azadirachta indica* (Meliaceae), known as “neem” possess phytochemicals used for anti-inflammatory properties. Isoprenoids (triterpenoids) are the major class of chemical constituents of *A. indica* (7) that constitutes more than 200 compounds in which azadirachtin (Figure 1A1) is a major compound, followed by nimbolide (Figure 1A2) and nimbin (Figure 1A3). Another plant, *Berberis aristata* (Berberidaceae), is known for its anti-inflammatory and immune-potentiating properties. The roots of *B. aristata* contain protoberberine alkaloids such as, berberine (Figure 1B4), oxyberberine, epiberberine, palmitine (Figure 1B5), and bis-isoquinoline alkaloids as its main constituents (8, 9).

Extraction of the crude plant material is a crucial step prior to their bio-evaluation. The most common extraction techniques used in laboratory are hot percolation, ultrasonication, and maceration (10). In all the extraction techniques, an important parameter is the type of solvent used. Non-polar solvents (e.g., hexane or petroleum ether) extracts sterols, terpenes; while, polar solvents such as methanol or ethanol help in extracting out polar compounds including alkaloids and polyphenols (11). Majority of research articles do not compare and correlate the biological efficacy of plant extracts with their extraction procedures. On the other hand, they mostly report the use of any one of the above techniques for preparation and bio-evaluation, which do not necessarily represent the true scenario. Thus, contextual optimization is needed to precisely understand the best extraction procedure to obtain the most effective biological results. The present study evaluates the chemotherapeutic efficacy of various extracts of *A. indica* seeds, *B. aristata* roots, and their combinations against cisplatin sensitive and resistant osteosarcoma cells. The study also highlights the comparison and correlation of the observed biological efficacy of above plant extracts with type of extraction techniques used.

## Materials and Methods

### Botanical Materials

Roots of *B. aristata* was collected from Mandi, Himachal Pradesh and seeds of *A. indica* was collected from the BITS-Pilani campus, Rajasthan. The plant materials were authenticated by a botanist in NIPER, SAS Nagar, India. Samples of the same have been deposited in the institute herbarium.

### Chemical and Reagents

Toluene, benzene, n-butanol, and ethylacetate were purchased from S. D. Fine Chemicals Ltd., Mumbai and acetic acid was purchased from Central Drug House Ltd., New Delhi. Anisaldehyde (4-methoxy benzaldehyde) was procured from Avra Synthesis Pvt. Ltd., Hyderabad.

### Extract Preparation

The plant materials were shed dried at room temperature and were processed properly into powder that was allowed to pass through BSS sieve #10. The powdered materials were divided into three parts (30 g each) and were subjected to three different extraction techniques namely soxhalation (SX, 24 h), ultrasonication (SO, 1 h), and maceration (MC, 72 h) using hexane and methanol. The extracts prepared were coded as BAH-SX, BAH-SO, BAH-MC, BAM-SX, BAM-SO, and BAM-MC for *B. aristata*, while for *A. indica*, they were coded as AIH-SX, AIH-SO, AIH-MC, AIM-SX, AIM-SO, and AIM-MC, respectively. The extracts were filtered and concentrated using rotary evaporator (10, 11). The extractive value of the extracts was calculated. The stock solutions for extracts were obtained in 10% tissue culture grade DMSO in PBS for a final concentration of 1 mg/ml. The stock solution was then diluted with media to form desired concentrations. The respective controls were formed by diluting a stock solution of 10% DMSO in PBS with media.

### Cell Culture

Human osteosarcoma (HOS) cell line was obtained from National Center for Cell Science (NCCS, Pune). The cell line was cultured in minimal essential medium (MEM; HiMedia Laboratories Pvt. Ltd.) supplemented with 10% fetal bovine serum (FBS; HiMedia Laboratories Pvt. Ltd.), 100 U/ml penicillin, and 100 µg/ml streptomycin (Invitrogen) (12). The cultures were maintained at 37°C in 5% CO_2_. Prior to treatment, cells were allowed to grow to 60–70% confluency and washed with PBS. Adherence was removed with 0.05% Trypsin–EDTA solution.

### Creation of Drug-Resistant Osteosarcoma Cells

Human osteosarcoma cell line resistant to cisplatin (HCR) was created following similar methods described in Palvai et al. (13). Briefly, HOS cells were exposed to repeated doses of very high concentration of cisplatin (1 mg/ml); surviving cells were selected and further sub-cultured. HCR cells were periodically checked for their resistance property by MTT assay and were maintained at 37°C in 5% CO_2_ under inhibitory concentration (IC_50_) cisplatin stress.

### *In Vitro* Cytotoxicity Assay

*In vitro* cytotoxicity was performed as described previously by Chowdhury et al. (12). Briefly, cells were cultured in 96 well plates. After 24 h, cells were treated with plant extracts for specific time periods. Following treatment, 20 µl of MTT [3-(4, 5-Dimethylthiazol-2-yl)-2, 5-Diphenyltetrazolium Bromide] (SRL) was added to each well along with 80 µl media and incubated for 4 h. Formazan crystals were solubilized in dimethyl sulfoxide (DMSO) and readings were obtained at 570 nm with a differential filter of 630 nm using Multiskan Microplate Spectrophotometer (Thermo Scientific). Percentage of viable cells was calculated using the following formula:

$$\text{Viability }\left(\text{\%}\right)=\frac{\text{mean absorbance value of drug-treated}}{\text{mean absorbance value of control}}\times 100.$$

DMSO control was parallelly run for each experiment.

### Estimation of Intracellular Reactive Oxygen Species (ROS)

Reactive oxygen species levels were measured using 2, 7-dichlorofluorescein diacetate (DCFH-DA) (Sigma), which measures intracellular generation of hydrogen peroxide, a procedure widely used for estimation of ROS. The DCFH-DA passively enters the cell, where it reacts with ROS to form the highly fluorescent compound dichlorofluorescein (DCF). Approximately, 0.3 × 10^4^ cells per well were seeded and treated with plant extracts for 24 h; the ROS scavenger *N*-acetyl cysteine (NAC, 5 mM) was added 2 h prior treatment wherever mentioned to inhibit ROS. Following exposure, cells were washed with PBS and then incubated in 100 µl of working solution of DCFH-DA (2 mM DCFH-DA stock solution was diluted to yield a 20 µM working solution) at 37°C for 30 min. The fluorescence was measured at 485 nm excitation and 530 nm emission using a microplate reader (Fluoroskan Ascent).

### Analysis of Apoptosis in Cultured Cells after Treatment

#### Annexin V/Propidium Iodide (PI) Staining

For determination of apoptosis, HOS cells were seeded in 6 cm dishes at a density of 1 × 10^6^ cells/dish. The following day, the cells were treated with specific plant extracts and incubated for 48 h. Thereafter, the cells were harvested, washed with PBS, and re-suspended in 500 µl of 1X binding buffer (BD BioSciences). To detect both early and late apoptotic cells, 4 µl of AnnexinV and 10 µl of PI were added to the cells in binding buffer, followed by incubation in dark for 20 min (12). The samples were then acquired using flow cytometer (Cytoflex, Beckmann Coulter) and analysis of acquired data was performed using CytExpert software. Percentage of apoptotic cells is represented through bar diagram.

#### Measurement of Caspase-3 Activity

Approximately, 1 × 10^4^ cells/well were seeded and exposed to plant extracts for 48 h. The activity of caspase-3 was measured using caspase-3 colorimetric protease assay kit (Invitrogen) following manufacturer’s protocol (12). Briefly, the cell lysate was collected in RIPA buffer and the concentration of protein was determined using Bradford assay. Equal amount (60 μg) of protein was added to microtiter plates with caspase-3 substrate (acetyl-Asp-Glu-Val-Asp p-nitroanilide, Ac-DEVD-pNA) and absorbance was read at 405 nm using a microplate reader (Start-fax 2100, Awareness Tech. Ltd.). The colorimetric assay is based on the hydrolysis of caspase-3 substrate by caspase-3 enzyme, resulting in the release of the p-nitroaniline (pNA) moiety. The concentration of the pNA released from the substrate was calculated from the absorbance values at 405 nm.

#### Analysis of DNA Fragmentation

A distinctive biochemical feature of apoptosis induction is the fragmentation of genomic DNA by specific nucleases like, caspase-activated DNAse (CAD). Activation of CAD by the caspase cascade in turn leads to cleavage of cellular DNA at inter-nucleosomal linker sites, generating short fragments of ~200 base pairs known as DNA ladders. One of the classical methods used to detect DNA ladders is to examine fragmented genomic DNA on an agarose gel. To analyze DNA ladder formation, HOS cells were seeded in 60 mm dishes at a density of 2 × 10^6^ cells/plate and treated with BAM-SX or AIM-SO for 48 h. Genomic DNA was extracted using Invitrogen Apoptotic DNA Ladder Detection Kit and ladder formation was analyzed in 1% agarose gel. A DNA marker was run parallel to the samples.

#### DAPI Staining of Nucleus

For detection of apoptotic nuclei by 4′-6-diamidino-2-phenylindole (DAPI) staining, cells were either untreated or treated with plant extracts for 48 h. Cells were grown on coverslips in 6 cm petridishes till confluency. Coverslips were then withdrawn, washed with 0.1 M PBS, and cells were fixed in methanol for 10 min at room temperature. Cells were then stained with DAPI (Sigma-Aldrich, USA) (1 mg/ml in PBS) for 20 min, washed twice with PBS, and the coverslips mounted on a glass slide. Nuclear morphology was then observed by fluorescence microscopy (Olympus, Japan). Apoptotic nuclei can be identified by the fragmented and or condensed chromatin of nuclear bodies, marked with white arrows.

### Flow Cytometric Analysis of Cell Cycle Phase Distribution

For DNA content analysis, HOS cells were seeded in 6 cm dishes at a density of 2 × 10^6^ cells/plate and grown overnight. After 48 h of treatment, the cells were harvested, washed with PBS, and centrifuged at 2,000 rpm for 5 min at 4°C. The pellet was then re-suspended in 100 µl of PBS and 900 µl of ice cold 70% ethanol, used as a fixative. The fixed cells were incubated at 4°C overnight. The next day, cells were centrifuged and the pellet was re-suspended in 450 µl PBS and 10 µl of PI (2 mg/ml) containing solution (12). The samples were then incubated in dark for 10 min followed by event acquisition using flow cytometer (Cytoflex, Beckmann Coulter) and analysis using CytExpert software. Percentage of cells in each phase of cell cycle was plotted and represented through bar diagram.

### Detection of Autophagy in Cultured Cells after Treatment

#### Monodansylcadaverine (MDC) Staining of Autophagic Vacuoles

The drug MDC, a specific autophago-lysosomal marker was used to analyze induction of autophagy after plant extract treatment (14, 15). For visualization of the autophagic vacuoles by fluorescence microscopy, HOS cells were seeded on cover slips and grown overnight. Following treatment, the cells were incubated for 10 min at 37°C with 0.05 mM MDC dissolved in PBS. The cover slips containing the cells were then washed with PBS and mounted with anti-fade mountant (containing DAPI). Intracellular MDC in the form of punctate dots were visualized using fluorescence microscopy. For, fluorimetric measurement, cells after treatment were labeled with MDC for 10 min, washed with PBS, and collected in 10 mM Tris–HCl (pH 8) containing 0.1% Triton X-100. Intracellular MDC was measured by fluorescence photometry (excitation 380 nm and emission 525 nm) in a microplate reader (Fluoroskan Ascent). An increase in MDC fluorescence upon treatment was expressed as fold change with respect to control.

#### Stable Transfection of GFP-RFP-LC3 and Fluorescent Detection

Plasmid GFP-RFP-LC3 was kindly provided by Dr. Sovan Sarkar (Birmingham Fellow, University of Birmingham). H1299 cells were cultured in six well plates and transfected with 2 µg of GFP-RFP-LC3 purified plasmid with Lipofectamine 3000 (Invitrogen, USA). Around 24 h after transfection, the cells were selected for transfection positivity by geneticin (600 µg/ml, Gibco) selection and were maintained for several days under geneticin pressure. Successful stable transfection of the plasmid was confirmed by fluorescent detection of LC3 in surviving cells through fluorescence microscopy (Olympus, Japan). For verification of autophagy induction, cells were treated with plant extracts and intracellular green and red fluorescence representing LC3 induction and accumulation were monitored and quantitated through fluorescence microscopy. By combining an acid-sensitive GFP with an acid-insensitive RFP in the vector, the autophagic flux is better visualized by imaging decreased GFP fluorescence, with a simultaneous increase in red fluorescence marking enhanced autophagic flux.

The LysoTracker probe (Thermo Fisher Scientific, USA), which consists of a fluorophore linked to a weak base, is a fluorescent acidotropic probe, which is often used for labeling and tracking acidic organelles in live cells. When cells were 60–70% confluent, the medium from the dish was replaced with pre-warmed (37°C), probe-containing medium. Cells were then incubated for 20 min, and the loading solution was replaced with fresh medium. The cells were then observed under a fluorescence microscope and the intensity of lysoTracker fluorescence was compared with untreated control.

### Bright Field Images

For bright field microscopic imaging, cells were cultured at density of 2 × 10^4^ cells/plate in 6 cm culture dishes, treated with phyto-extracts, and then images were captured using Olympus (CKX41) microscope at 20 × magnification.

### Phytochemical Analysis

The active extracts from each plant were selected for phytochemical analysis using CAMAG high performance thin layer chromatography (HPTLC) instruments equipped with winCAT software. Extracts were dried properly and re-dissolved in methanol as 1 mg/ml stock solution. TLC plates with 0.2 mm pre-coated silica gel 60F_254_ (Merck, Germany) of 5 × 10 cm were taken. The extract samples were spotted using Linomat 5 automated sample spotter (CAMAG) using 100 µl syringe (Hamilton, Switzerland). Sample solution (5 µl) was spotted as bands of 6 mm width with the syringe with 5 mm distance between each spot. The spotted plates were developed in glass twin trough chamber (10 cm × 10 cm, CAMAG). n-butanol: acetic acid: water (14:3:4 v/v/v) was used as mobile phase for BAM-SX (16, 17), while benzene: ethyl acetate (3:7 v/v) (18) and hexane:ethyl acetate:acetic acid (6:4:0.2) were used as mobile phase for AIM-SO (18–20) to identify compounds like azadirachtin, nimbin, and nimbolide. The length of the chromatogram was 80 mm from application position. After development, the TLC plates were dried using hot plate and scanned densitometrically at 254 and 366 nm. The image of the developed TLC plate was captured using TLC Visualizer (CAMAG) under 254 and 366 nm. The developed plate of AIM-SO was derivatized using anisaldehyde reagent followed by heating the plate at 105°C for 5 min. The derivatized plate was scanned at 560 nm and image was captured under white light using visualizer. The following scan conditions were applied: slit width: 4 mm × 0.10 mm, distance from Y-position: 10 mm and distance from X-position: 10 mm. Samples were run in triplicate. Experimental conditions: temperature 25°C ± 2; relative humidity 50%.

### Statistical Analysis

Tukey tests was used as a follow up to one-way or two-way ANOVA to compare every mean to a control mean and every mean with every other mean using Graph Pad Prism software version 5.0. The Bonferroni method was used to analyze multiple comparisons using Graph Pad Prism software version 5.0. The tests compute a confidence interval for the difference between the two means. The (*) in figures denotes a significant change with reference to compared sample at 95% confidence level.

## Results

### Effect of Extraction Techniques on Cytotoxicity of Methanolic Extract of *B. aristata* and *A. indica*

**Figure 2:**
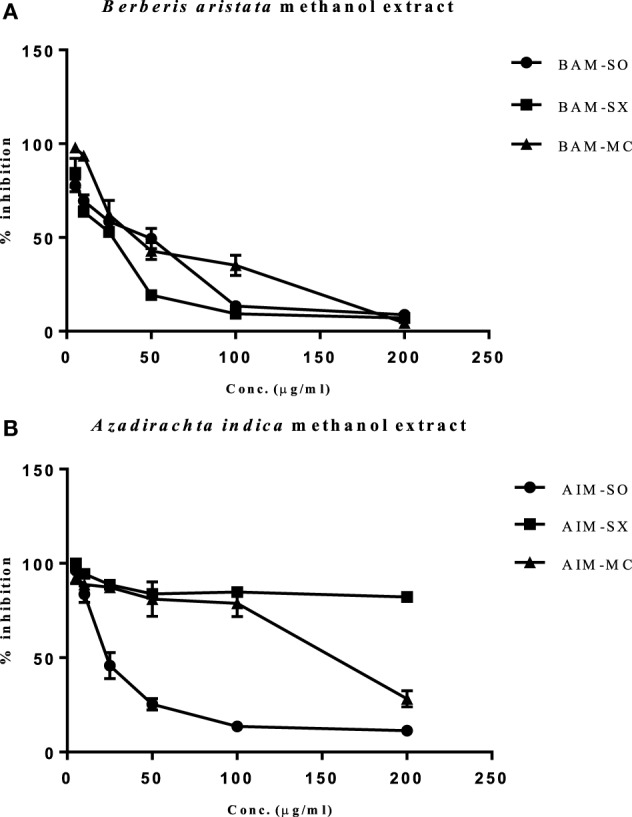
Evaluation of cytotoxic effect of BAM and AIM on human osteosarcoma (HOS) cells. Cells were treated with sonication (BAM-SO), soxhlation (BAM-SX), and maceration (BAM-MC) **(A)** extracts for 48 h and cell viability was analyzed through MTT assay. Cells were treated with sonication (AIM-SO), soxhlation (AIM-SX), and maceration (AIM-MC) **(B)** extracts for 48 h and cell viability was analyzed through MTT assay. The cytotoxic effect on HOS is represented through curved diagram.

In our study, the various extracts of *B. aristata* and *A. indica* were tested against osteosarcoma cells (HOS), the most common primary malignant bone tumor among children and adolescents, for their cytotoxic potential. BAM-SX (IC_50_ 27.82 µg/ml) and BAM-SO (IC_50_ 29.26 µg/ml) showed considerably more cytotoxicity than BAM-MC (IC_50_ 46.8 µg/ml), respectively (Figure 2A). A possible reason justifying the increased cytotoxicity of BAM-SX could be due to more extractive value of BAM-SX (7.26 g) than BAM-SO (6.52 g) and BAM-MC (3.28 g). Soxhlation performed for 24 h is probably enough to extract out active constituents into the solvents. Furthermore, the underlying reason behind the comparatively less cytotoxicity of BAM-SO could be associated with ultrasound energy mediated deleterious effects on the active compounds. However, as the extractive value of BAM-SX and BAM-SO is more or less similar, hence, there is not much difference in the bioactivity of the extract. However, in contrary, AIM-SO showed enhanced cytotoxicity (IC_50_ value of 28.65 µg/ml) in HOS cells than AIM-SX or MC (Figure 2B). Probably, the amount of active constituents was more in AIM-SO compared to AIM-SX and AIM-MC. It is speculated that ultrasound waves can help the solvent to penetrate into the cell wall of the plant material extracting out more compounds for cytotoxicity. Since BAM-SX and AIM-SO showed maximum cytotoxicity, for further studies, BAM-SX and AIM-SO were selected.

### Induction of ROS Precedes Cell Death in AIM-SO Treated and BAM-SX-Treated Cells

**Figure 3:**
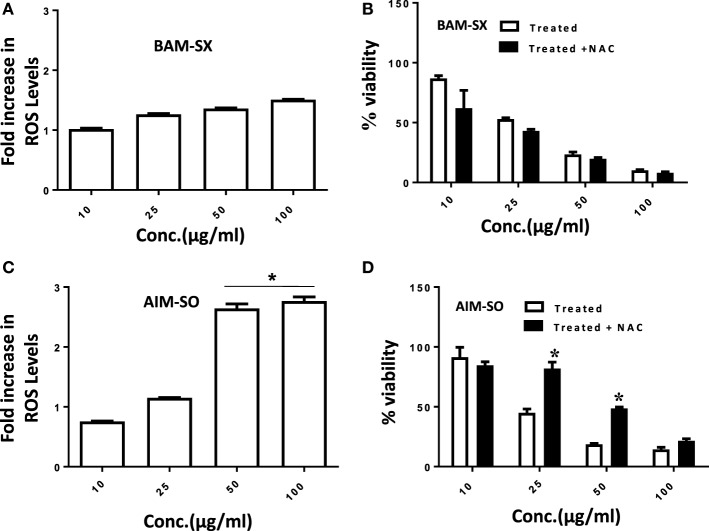
Estimation of reactive oxygen species (ROS) levels upon BAM-SX and AIM-SO treatment in human osteosarcoma (HOS) cells. **(A)** HOS cells were exposed to BAM-SX at different concentrations for 24 h. NAC (5 mM) was applied 1 h prior to treatment wherever mentioned. Fold change in ROS levels as measured by DCFH-DA is represented; untreated control was considered as arbitrary unit “1.” **(B)** MTT assay was performed to check cell viability following exposure of HOS cells to BAM-SX. **(C)** HOS cells were exposed to AIM-SO at different concentrations for 24 h. Fold change in ROS levels as measured by DCFH-DA is represented; untreated control was considered as arbitrary unit “1.” **(D)** MTT assay was performed to check cell viability following exposure of HOS cells to AIM-SO.

Cancer cells are known to generate moderate to high levels of ROS to aid their need for enhanced proliferation, migration, and metastasis (21, 22). However, conversely, increasing the level of ROS has often been utilized as a strategy to tip the balance of cancer cells toward cell death (21). Under the above circumstances, the generation of enhanced ROS can play a pivotal role in the initiation of apoptosis. Existing literature hints toward the prospective potential of phytochemicals in altering cellular ROS levels and induction of anticancerous activity (1, 2). However, the potential of natural extracts of BAM and AIM for ROS generation in osteosarcoma has not been evaluated before. A more than 2.5-fold accumulation of ROS was observed in cells treated with AIM-SO compared to untreated control after 24 h of treatment, as measured fluorometrically through H2DCF-DA assay, which acts as a marker for ROS (Figure 3A). However, BAM-SX failed to induce a significant generation of ROS at the time point studied (Figure 3B). ROS generation however, can be dependent on and resultant of the cell type studied, and the extraction procedure involved. To further understand the role of ROS, we inhibited intracellular ROS with NAC. Pretreatment of cells with the ROS scavenger NAC significantly increased cell viability in AIM-SO treated cells, but failed to show a significant effect in BAM-SX treated cells (Figures 3C,D). From the above experiments, it can be concluded that AIM-SO induces ROS-dependent cytotoxicity, while, BAM-SX stimulates ROS-independent cell death in the HOS cells studied.

### BAM-SX and AIM-SO Induce Sub-G_1_ Population of Cells, Caspase Activity, and Apoptotic Cell Death in HOS Cells

**Figure 4:**
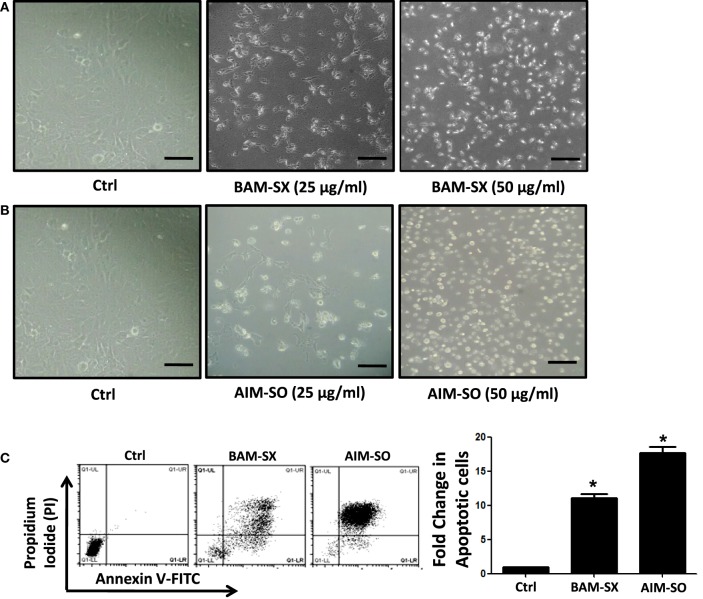
BAM-SX or AIM-SO induces apoptosis in human osteosarcoma (HOS) cells. **(A,B)** Phase contrast images of HOS cells were captured after treatment with phyto-extracts—BAM-SX and AIM-SO for 48 h. Scale bar = 100 µm. **(C)** *Indicates statistical significance in the fold change in apoptotic cells.

**Figure 5:**
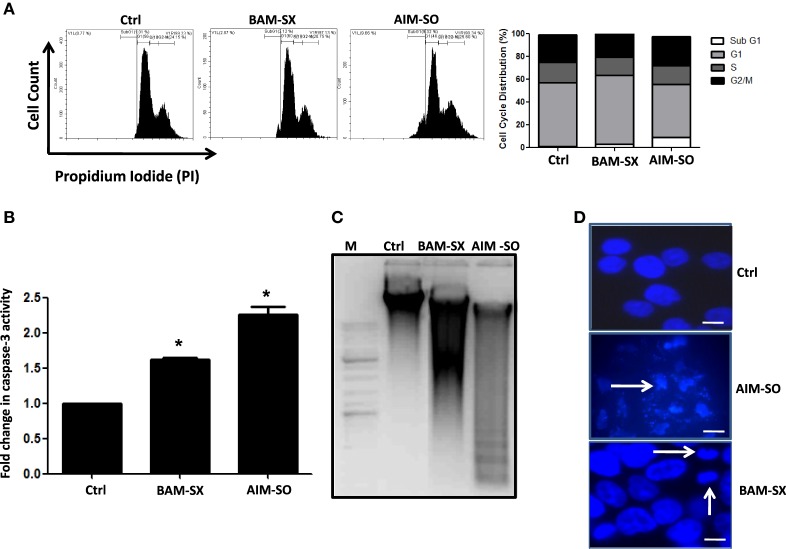
Presence of apoptotic cells was evaluated by AnnexinV-PI staining after treatment of human osteosarcoma (HOS) cells with BAM-SX or AIM-SO at IC50 dose for 48 h through flow cytometry. In the representative figure, cells in lower right and upper right quadrant represent early and late apoptosis cells, respectively. A fold increase in apoptotic cells was calculated with respect to untreated control (taken as arbitrary unit “1”) and is represented through bar diagram. **(A)** Number of cells in each phase of the cell cycle was analyzed by propidium iodide (PI) staining of HOS cells exposed to BAM-SX or AIM-SO at IC50 dose for 48 h. A representative image of flow cytometric analysis is provided. Percentage of cells in each phase of cell cycle- sub-G1, G1, S, and G2 is depicted in the figure. Fold change in sub-G1 cell population with respect to untreated control (taken as arbitrary unit “1”) is represented through a bar diagram. *Indicates the significant difference of the cells in the sub-G1 phase compared to untreated control cells. **(B)** Fold change in caspase-3 enzyme activity was measured following BAM-SX and AIM-SO treatment for 24 h. Level of caspase-3 activity in untreated control was taken as arbitrary unit “1.” **(C)** Presence of DNA ladder evident through formation of a smear in gel electrophoresis is presented with a representative figure. HOS cells were treated with BAM-SX or AIM-SO at IC50 dose for 48 h, DNA was extracted and run on 1% agarose gel. A DNA marker was run alongside the samples. **(D)** Cells were either untreated or treated with plant extracts for 48 h and then stained with DAPI. Nuclear fragmentation or condensation posttreatment is marked with white arrows.

Phytochemicals are known to trigger apoptosis to inhibit cancer progression and such apoptotic cell death signaling is often elicited post ROS accumulation in cancer cells (1, 2). Previous studies have reported that nimbolide or berberine can induce apoptotic cascade *via* ROS production in various cancer cell types like colon and human choriocarcinoma cells (23–26). However, the potential of BAM and AIM in induction of apoptosis in osteosarcoma cells, a highly malignant cancer type with inherent resistant properties has not been elucidated yet. Phase contrast images of cells treated with the phytoextracts showed distinct rounding up of cells posttreatment, indicative of apoptotic cells (Figures 4A,B). We were, therefore, interested to analyze induction of apoptosis through other methods. Apoptosis induction in HOS cells, posttreatment, was measured through flow cytometry using AnnexinV-FITC-PI. A significant increase in apoptotic cells was observed in cells independently treated with BAM-SX and AIM-SO (Figure 4C). As the extracts showed significant growth inhibitory effect on HOS cells, we determined the possible inhibitory effect of BAM-SX and AIM-SO on the cell cycle progression of HOS cells. As summarized in Figure 5A, treatment of HOS cells with BAM-SX for 48 h resulted in a significantly higher number of cells in the sub-G_1_ phase (12%) of the cell cycle compared to non-BAM-SX-treated control. There was a concomitant reduction in the number of cells in G_2_-M phase of the cell cycle. Similar, but, significantly more pronounced (24%) results were obtained in cells treated with AIM-SO (Figure 5A). These data suggest that inhibition of cell proliferation or induction of cell death in HOS cells by BAM-SX and AIM-SO is associated with the disarray of cell cycle profile of HOS cells. Stimulation of apoptosis is known to release cytochrome c from mitochondria and lead to the activation of apaf-1 (apoptosome), which in turn cleaves the pro-enzyme caspase-9 into its active form; activated caspase-9 then activates executioner caspases, like caspase-3 ultimately leading to cell death. We evaluated caspase-3 activity through ELISA in the extract treated cells (Figure 5B). In continuation to the results obtained above, BAM-SX and AIM-SO were found to induce caspase-3-dependent apoptosis in HOS cells. Also, DNA fragmentation is often considered as a hallmark of apoptosis. As evident in Figure 5C, a smear indicative of fragmented DNA post apoptosis was observed in cells treated with the plant extracts. Furthermore, analysis of nuclear morphology by DAPI staining post extract treatment revealed the presence of condensed and fragmented nucleus in plant extract treated cells indicative of apoptosis induction (Figure 5D). Our data indicate that treatment of HOS cells with the plant extracts results in G_1_ arrest and associated induction of apoptosis.

### Autophagy Acts As Protective Strategy against the Cytotoxic Effects of BAM-SX and AIM-SO in HOS Cells

**Figure 6:**
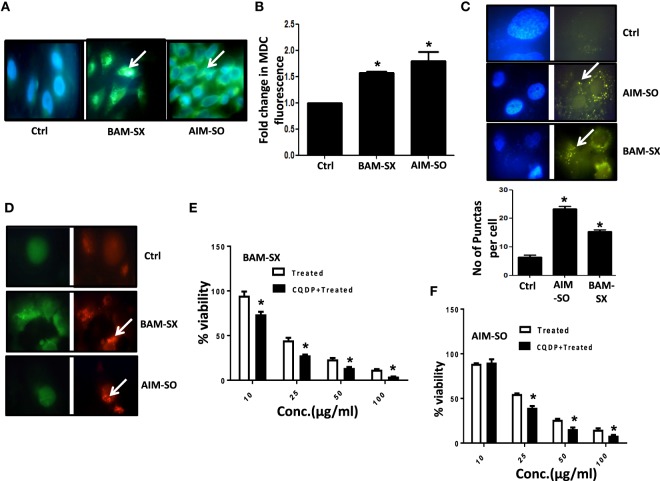
The phytoextracts induce autophagy as a survival strategy in human osteosarcoma (HOS) cells. **(A)** For detection of autophagy, monodansylcadaverine (MDC) fluorescence staining was performed after 48 h of BAM-SX or AIM-SO treatment at IC_50_ dose in HOS cells and green punctate dots indicative of autophagosomes were monitored by fluorescence microscopy. The scale bar represents 100 µm. **(B)** Fluorimetric estimation of MDC fluorescence was performed following BAM-SX or AIM-SO treatment at IC_50_ dose for 48 h. **(C)** Cells were treated with BAM-SX or AIM-SO at IC_50_ dose for 48 h and then stained with LysoTracker dye. Green dots representing the lysosomes were counted between control and treated cells and represented as bar diagram. **(D)** Cells stably transfected with GFP-RFP LC3 vector were exposed to IC_50_ dose of BAM-SX or AIM-SO for 48 h and then relative green and red fluorescence was quantitated by fluorescence microscopy. White arrows indicate enhanced red fluorescence in treated cells. **(E,F)** Cytotoxic effect of BAM-SX or AIM-SO treatment at various doses in HOS cell was measured by MTT assay after 48 h of treatment in presence or absence of autophagy inhibitor [10 µM, chloroquine diphosphate (CQDP)]. The inhibitor was added 2 h before treatment.

Autophagy is an essential cellular phenomenon by which cells maintain intracellular homeostasis through degradation and recycling of proteins and organelles through fusion with lysosomes. The role of autophagy in cancer has been controversially discussed (27). It has been known to both support or suppress tumorigenesis more in a context-dependent manner (28). In this regard, berberine has been previously demonstrated to induce autophagy and mitochondrial apoptosis in human hepatic carcinoma cells (29); here, autophagy provided a pro-death signal, as inhibition of autophagy with 3-methyl adenine (3-MA) reduced cell death. Furthermore, Wang et al. showed that berberine can lead to high autophagic flux resulting in reduction of invasive properties and proliferative potential of glioblastoma cells (30). However, in contrary, Subramani et al. reported that nimbolide can potentiate autophagy in pancreatic cancer cells as an adaptive stress response to extend cell viability rather than to initiate apoptosis/cell death (31). Thus, autophagy can act either as a cyto-protective or as a cytotoxic phenomenon, and hence it is important to understand its role in each context independently. In our study, we observed that on treatment with BAM-SX and AIM-SO, HOS cells upregulate autophagy. Autophagy was estimated by quantitation of MDC fluorescence both microscopically and fluorimetrically (Figures 6A,B). A significant increase in MDC fluorescence reflective of green dots in perinuclear region was observed in cells 48 h after treatment with the phytochemicals (Figures 6A,B). A LysoTracker probe was also used to label and detect acidic organelles, which mainly consist of lysosomes. In our study, we observed increased green puntate dots with stronger fluorescence intensity in cells treated with the natural extracts indicating an enhanced lysosomal number and probable function in the treated cells (Figure 6C). H1299 Cells were further stably transfected with GFP-RFP-LC3 plasmids, and we observed that posttreatment, there was a significant increase in red fluorescent punctate dots indicative of enhanced autophagy after treatment (Figure 6D). In corroboration to above, interestingly, autophagy inhibitor chloroquine di-phosphate (CQDP) induced enhanced cell death in the phyto-extract treated cells. Cells were treated with CQDP, 2 h prior to treatment with BAM-SX and AIM-SO and cell viability was estimated using MTT assay (Figures 6E,F). Our results are indicative of the fact that autophagy is triggered post BAM-SX or AIM-SO treatment in HOS cells, which facilitate the cells to overcome the cytotoxic effect; hence autophagy here operates as a cytoprotective phenomenon.

### Combination Study of BAM-SX and AIM-SO

Effect based strategy is a method in which combined effect of two drugs can be directly compared with the effect of individual drugs. This strategy depends on the combination index (CI). It is calculated to show the positive drug combination effect occurs when the observed combination effect (E_AB_) is greater than the expected additive effect given by the sum of the individual effects (E_A_ + E_B_).

$$\text{CI}=\frac{\left({\text{E}}_{\text{A}}+{\text{E}}_{\text{B}}\right)}{{\text{E}}_{\text{AB}}}.$$

**Figure 7:**
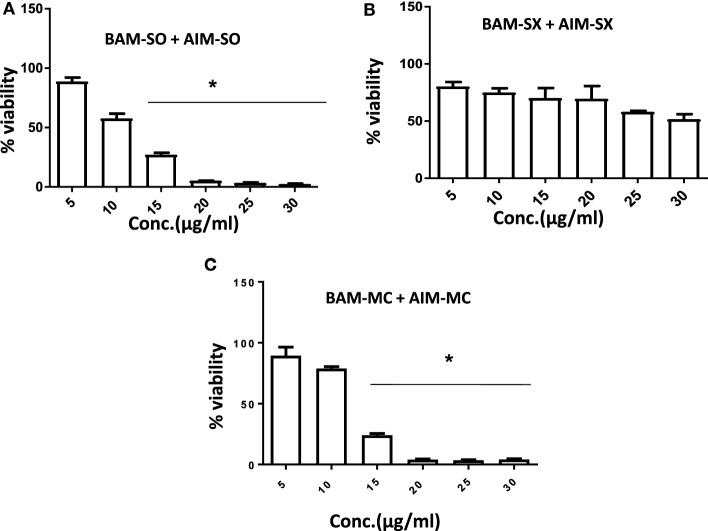
Phyto-extracts in combination induce synergism or antagonism cytotoxicity in human osteosarcoma cells. Cell viability was analyzed by MTT assay after 48 h of treatment with various combination of plant extracts BAM-SO and AIM-SO **(A)**, BAM-SX and AIM-SX **(B)**, BAM-MC or AIM-MC **(C)**.

**Figure 8:**
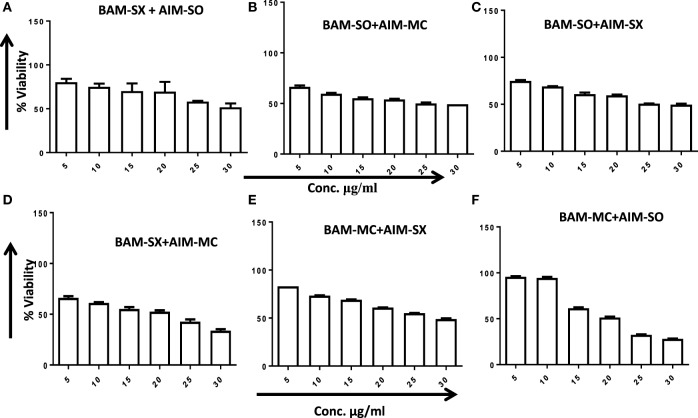
Phyto-extracts in combination induce synergism or antagonism cytotoxicity in human osteosarcoma cells. Cell viability was analyzed by MTT assay after 48 h of treatment with various combination of plant extracts BAM-SX and AIM-SO **(A)**, BAM-SO and AIM-MC **(B)**, BAM-SO and AIM-SX **(C)**, BAM-SX and AIM-MC **(D)**, BAM-MC and AIM-SX **(E)**, BAM-MC and AIM-SO **(F)**.

**Figure 9:**
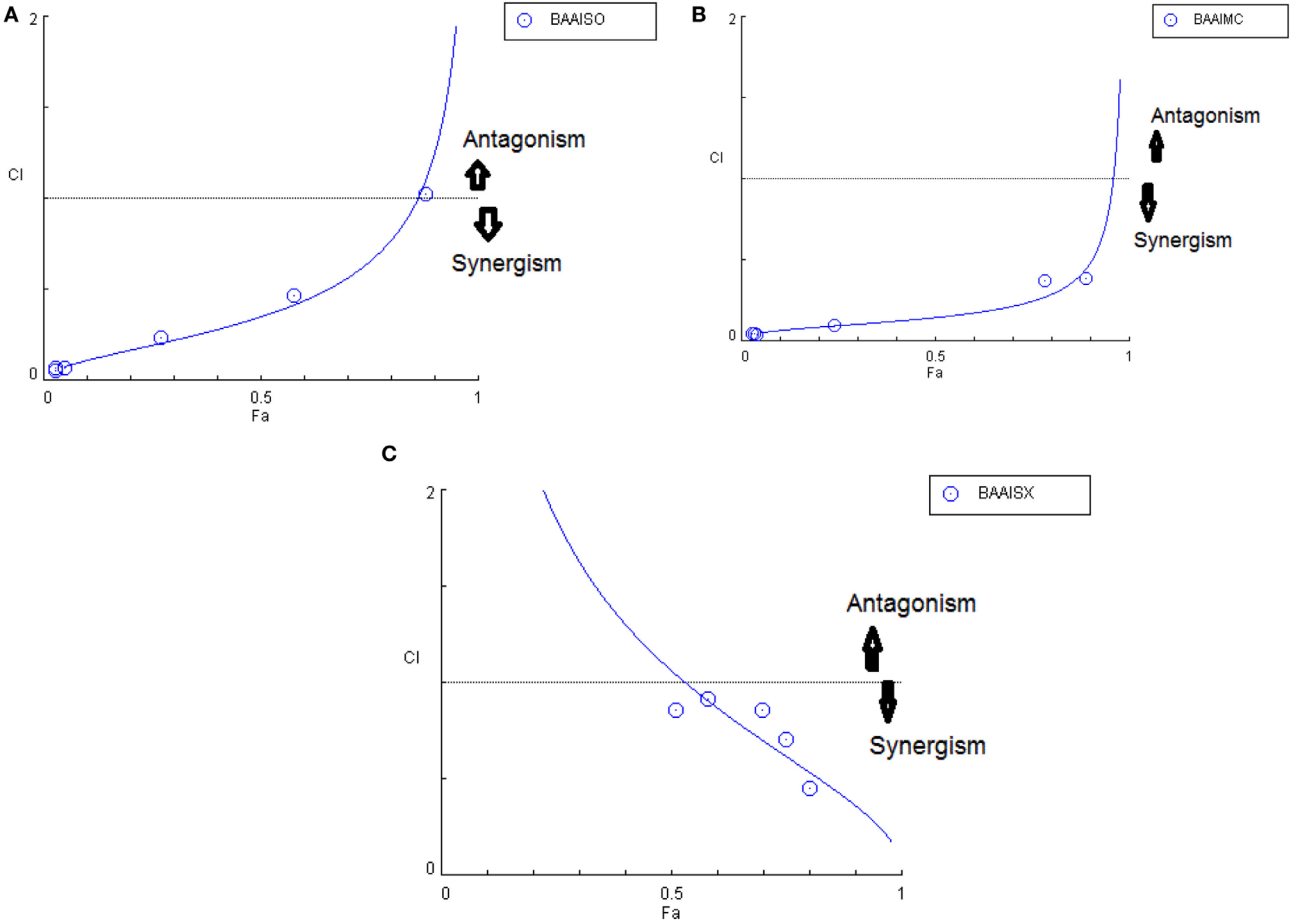
Combination index plot (using Composyn software) for BAM-SO and AIM-SO (BAAISO) **(A)**, BAM-MC and AIM-MC (BAAIMC) **(B)**, BAM-SX and AIM-SX (BAAISX) **(C)**.

**Figure 10:**
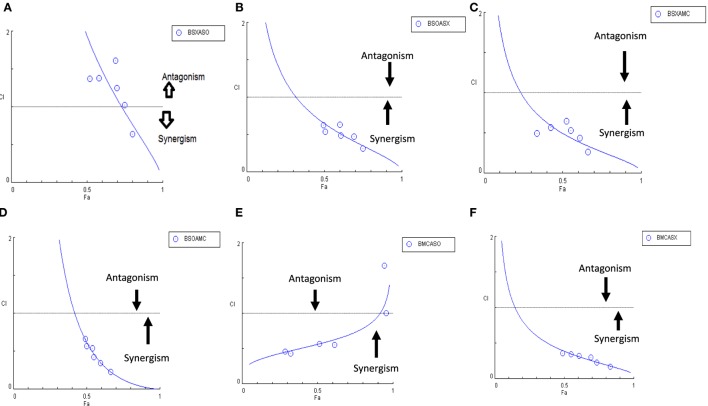
Combination index plot (using Composyn software) for BAM-SX and AIM-SO (BSXASO) **(A)**, BAM-SO and AIM-SX (BSOASX) **(B)**, BAM-SX and AIM-MC (BSXAMC) **(C)**, BAM-SO and AIM-MC (BSOAMC) **(D)**, BAM-MC and AIM-SX (BMCASX) **(E)**, and BAM-MC and AIM-SO (BMCASO) **(F)**.

Another advantage of this method is that it provides complement to the algebraic analysis with an accepted graphical approach (32). The combined effect of the two cytotoxic extracts in the ratio of 1:1 was observed for 48 h in pairs of sonicated extracts, i.e., BAM-SO and AIM-SO (BAAISO) and macerated extracts, i.e., BAM-MC and AIM-MC (BAAIMC) with IC_50_ of 10.36 and 11.27 µg/ml, respectively (Figures 7A,C). Negligible cytotoxity was observed even after 48 h when the combination with pair of soxhlated extracts, i.e., BAM-SX and AIM-SX (BAAISX) in 1:1 ratio was performed (Figure 7B). The combination studies were performed using cross technique extracts of BAM and AIM, i.e., BAM-SO and AIM-SX (BSOASX), BAM-SX and AIM-MC (BSXAMC), BAM-SO and AIM-MC (BSOAMC), BAM-MC and AIM-SX (BMCASX), and BAM-MC and AIM-SO (BMCASO), which showed cytotoxity below 30 µg/ml in the ratio of 1:1 on HOS cell lines for 48 h, but the combination of BAM-SX and AIM-SO (BSXASO) in the same conditions showed IC_50_ of 53.37 µg/ml (Figures 8A–F). Effect-based strategy was applied using software Compusyn version 1. The results showed that BAAISO showed dose-dependent synergism as increase in concentration of BAM-SO and AIM-SO in the ratio of 1:1 (Figure 9A). On the other hand, BAAIMC showed synergism in all the doses of BAM-MC and AIM-MC in 1:1 ratio (Figure 9B). BAAISX, combination of BAM-SX and AIM-SX showed antagonistic effect in the same ratio (Figure 9C). Inter technique extract combination showed synergism (Figure 10B–F) in a dose-dependent manner except BSXASO, which showed antagonism as CI is more than 1 in all doses of BAM-SX and AIM-SO in 1:1 ratio (Figure 10A) (Table 1). As BAAISO was found to be most potent against HOS cell lines compared to the other combinations, it was further tested on HCR cell lines.

**Table 1:** Combination index (CI) of the tested combinations in human osteosarcoma cells at dose of 25 µg/ml.

Sl no.	Combinations	Dose (μg/ml)	CI
1.	BAAISO	25	0.054
2.	BAAIMC	25	0.045
3.	BAAISX^a^	25	0.913
4.	BSXASO^a^	25	1.384
5.	BSOASX	25	0.542
6.	BSXAMC	25	0.571
7.	BSOAMC	25	0.572
8.	BMCASX	25	0.334
9.	BMCASO	25	0.429

*^a^Indicates that the combination shows antagonistic activity*.

### BAM-SX and AIM-SO Sensitizes Resistant HOS Cells

**Figure 11:**
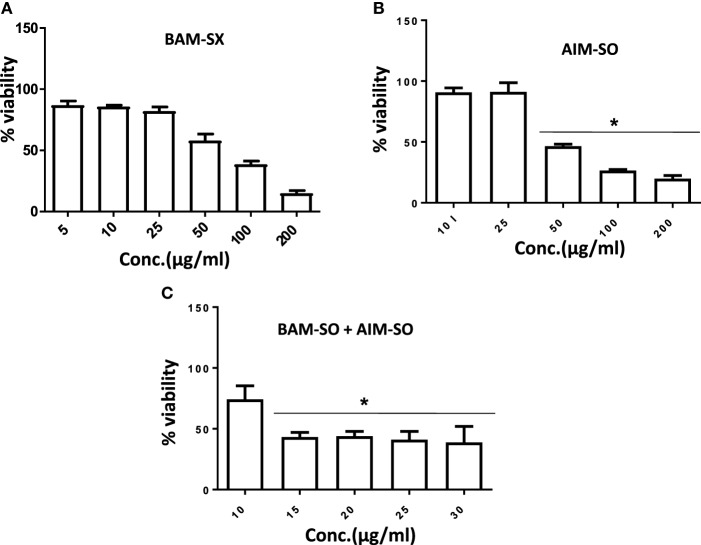
Phyto-extracts alone or in combination induce cytotoxicity in cisplatin resistant human osteosarcoma cells (HCR). Cell viability was analyzed by MTT assay after 48 h of treatment with independent or combination of plant extracts BAM-SX **(A)**, AIM-SO **(B)**, and BAM-SX plus AIM-SO **(C)**.

Human osteosarcoma is the most prevalent malignant bone tumor with a 5-year survival rate of as low as 20%. Even with an aggressive treatment regimen, disease recurrence is almost always invariable in osteosarcoma. Thus, therapy resistance remains a major concern, which needs to be promptly addressed (33, 34). We, therefore, derived a cisplatin resistant *in vitro* model from the parental HOS cells following similar procedure described in Palvai et al. (13) and tested for the cytotoxic efficacy of BAM-SX and AIM-SO in the resistant cells. While creating the resistance model, the HOS cells were exposed to high dose of cisplatin followed by clonal selection of surviving cells post shock. This process was repeated several times to derive HOS cells that showed decreased sensitivity to cisplatin. These cells were termed as HCR, representing cells resistant to cisplatin. The concentration of cisplatin at which 50% of HOS cells died was 35 µM, while an increased viability (80%) was obtained in HCR cells at similar concentration of cisplatin, as estimated through MTT assay. To maintain clinical relevance, a resistance model showing more than twofold resistance to cisplatin was not created for this study. BAM-SX and AIM-SO were able to sensitize the resistant HCR cells with an IC_50_ value of 80.87 and 43.93 µg/ml, respectively, obtained only after 24 h of treatment (Figures 11A,B). As evident from the values, AIM-SO was found to be significantly more cytotoxic than BAM-SX. However, the combination of BAM-SO and AIM-SO (1:1) showed significantly increased cytotoxicity with an IC_50_ value of 17.34 µg/ml (Figure 11C). The combinatorial treatment of BAM-SX and AIM-SO failed to impart enhanced cytotoxicity compared to their treatment when administered independently. Our results clearly indicate that the extracts tested in this study are potent enough to effectively sensitize cisplatin resistant cells.

### Phytochemical Analysis

**Figure 12:**
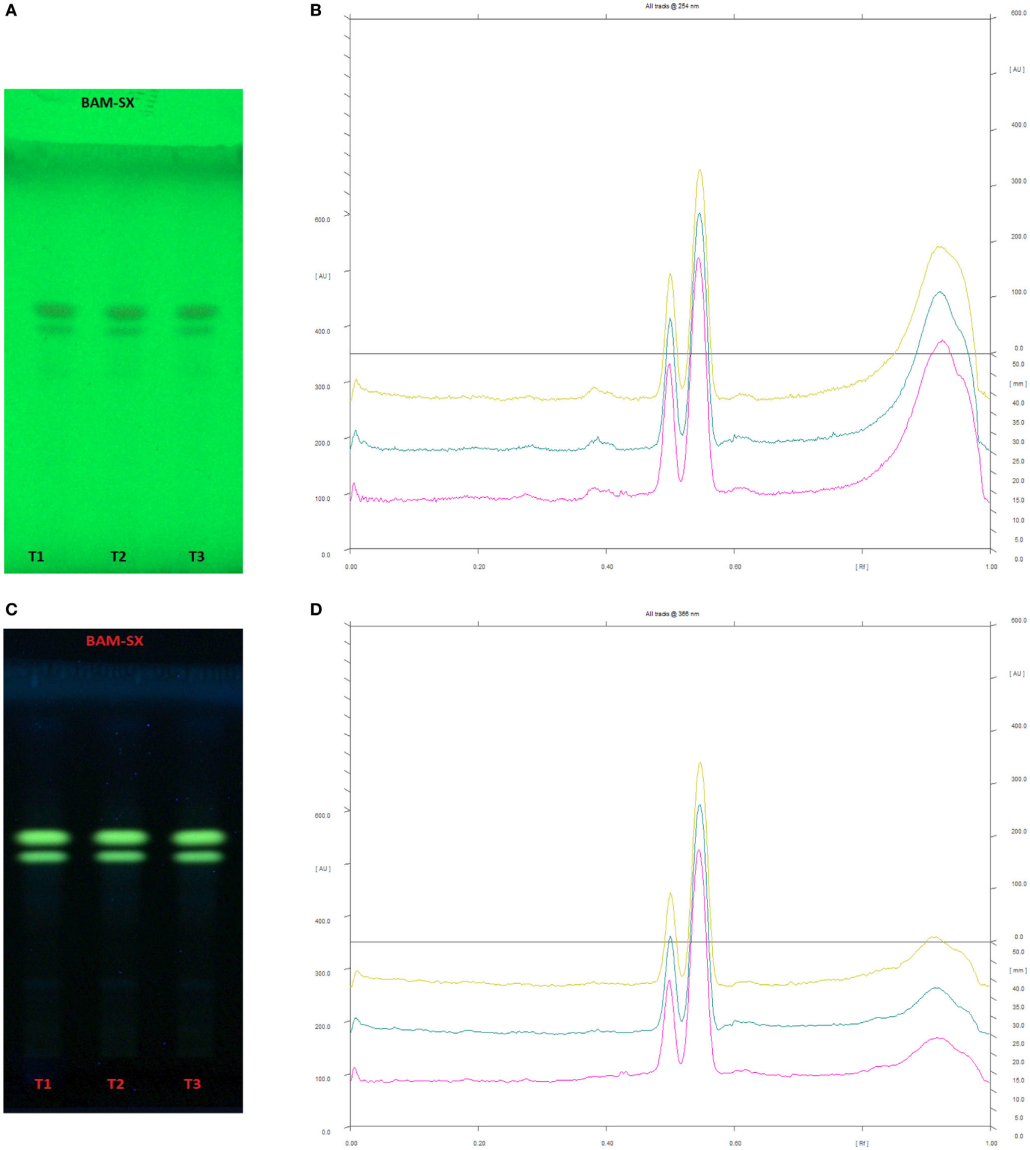
High performance thin layer chromatography profiling of *Berberis aristata* (methanolic extraction using Soxhlation -BAM-SX) using the solvent system as n-butanol:acetic acid:water (14:3:4); berberine (0.55); almatine (0.50). **(A)** The developed plate at 254 nm, **(B)** chromatogram of the developed plate after scanning at 254 nm, **(C)** developed plate at 366 nm, **(D)** chromatogram of the developed plate after scanning at 366 nm.

Since BAM-SX and AIM-SO were found to induce apoptosis in HOS cells, hence, a HPTLC-based phytochemical analysis was carried out in order to understand the phytochemical profile of these extracts. There are various HPTLC methods reported for analysis of *B. aristata* and *A. indica*. However, as variation is found to occur in botanical samples due to different geographic locations and climatic conditions, identification of compounds is necessary. Reported composition of mobile phase (*n*-butanol:acetic acid:water; 14:3:4v/v/v) was used for BAM-SX, which showed berberine as the major components (R_f_ 0.55) followed by palmatine (R_f_ 0.50) at 254 and 366 nm (Figure 12). Berberine has been reported to affect activities of enzymes such as N-acetyltransferase, cyclooxygenase-2, and topoisomerase, by modifying gene/protein expression. These actions along with the ROS production, mitochondrial transmembrane potential, and NF-κβ activation have been found to potentiate antiproliferative and proapoptotic effects of *B. aristata* (24, 29, 35). Palmatine has also been shown to exhibit cytotoxicity in mice by selectively targeting DNA topoisomerase I (36, 37).

**Figure 13:**
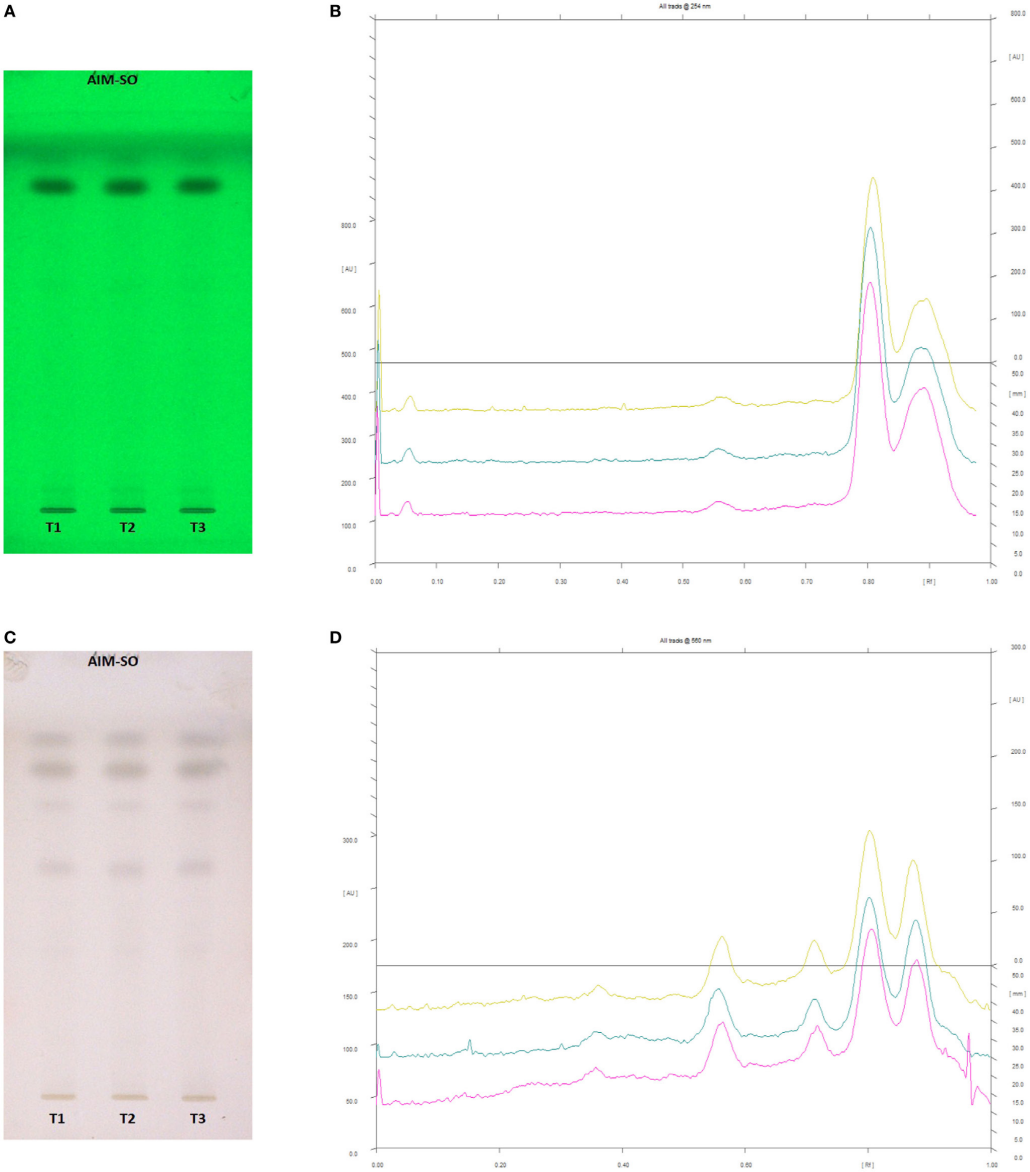
High performance thin layer chromatography profiling of *Azadirachta indica* (methanolic extraction using Sonication—AIM-SO) using the solvent system as benzene:ethyl acetate (3:7); azadirachtin A (0.36); azadirachtin B (0.56); nimbin (0.80). **(A)** The developed plate at 254 nm, **(B)** chromatogram of the developed plate after scanning at 254 nm, **(C)** developed plate after derivatizing with anisaldehyde reagent and dried at 105°C at 560 nm, **(D)** chromatogram of the derivatized plate after scanning at 560 nm.

**Figure 14:**
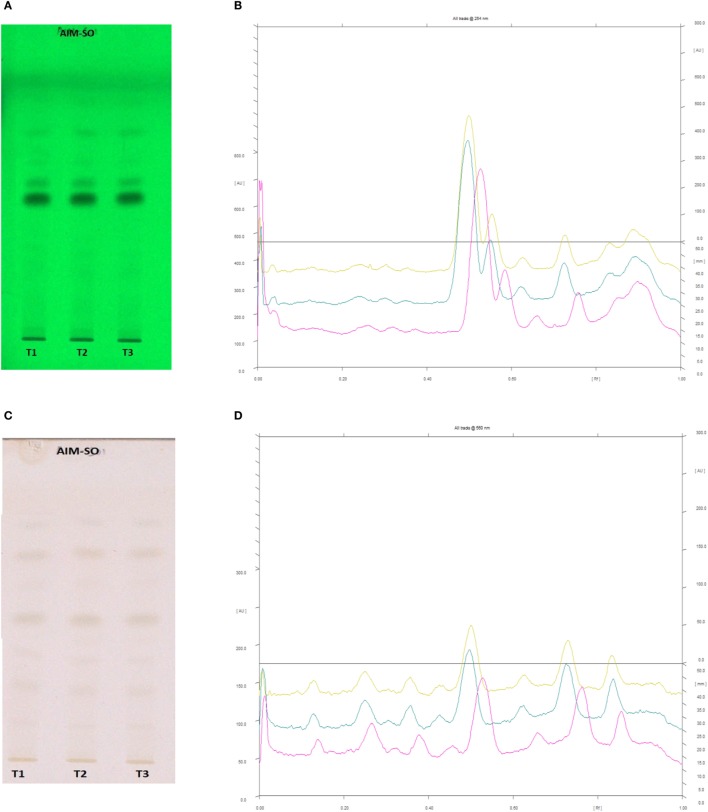
High performance thin layer chromatography profiling of *Azadirachta indica* (methanolic extraction using Sonication—AIM-SO) using the solvent system as hexane:ethyl acetate:acetic acid (6:4:0.2); nimbolide (0.43). **(A)** The developed plate at 254 nm, **(B)** chromatogram of the developed plate after scanning at 254 nm, **(C)** developed plate after derivatizing with anisaldehyde reagent and dried at 105°C at 560 nm, **(D)** chromatogram of the derivatized plate after scanning at 560 nm.

In case of AIM-SO, two reported solvent systems were used. The first solvent system was used to identify compounds like azadirchtin A (R_f_ 0.36), azadirachtin B (R_f_ 0.56), and nimbin (R_f_ 0.80) in benzene:ethyl acetate (3:7). The other solvent system was used to identify nimbolide (R_f_ 0.43) using hexane:ethyl acetate:acetic acid (6:4:0.2). However, very few phytochemicals were visible as bands at 254 and 366 nm, clearly indicating poorly absorbing chromophores in the extract. Hence, the developed TLC plate was derivatized with Anisaldehyde reagent. The derivatized plate was scanned at 560 nm. The derivatization resulted in detection of compounds that were identified by their respective R_f_ values (Figures 13 and 14). Nimbolide has shown enhanced cytotoxic and apoptotic inducing potential. It does this by increasing the generation of ROS and downregulation of proliferating cell-nuclear antigen leading to apoptosis (23, 31, 38). Azadirachtin on the other hand has been found to decreases the over expression of cyclin-E, leading to the shortening of G_1_ phase of cell cycle and reducing the production of cyclin-A, a protein responsible for cell division, which leads to apoptosis (8, 39).

## Conclusion

In spite of enormous progress in the last decade, in the field of cancer therapeutics, tumor relapse and drug refractoriness has been highly prevalent and has not yet been addressed effectively (4). Natural products and herbal therapies have demonstrated promise and prospect in prevention and treatment of various cancers, but, their true potential as remedies toward aggressive cancers has been underexplored (1, 2). Hence, though the usefulness of plant extracts in attenuating cancer progression has been authenticated by the increasing number of commercially available anticancer drugs detailed studies illustrating their effectiveness against inherently resistant cancer cells has been elusive. In this study, we have evaluated the anticancerous efficacy of BAM and AIM against both drug sensitive and resistant osteosarcoma (HOS) cell types. Cells were treated with plant extracts derived through various extraction procedures to compare their potential effectiveness against the above cell types. We report that BAM extracts prepared by soxhalation technique and AIM extracts prepared by sonication were the most effective in inducing apoptosis in HOS cells. The HOS cells are reported to be inherently resistant and hence cytotoxicity of the herbal compounds has enormous significance from clinical perspective. Additionally, the extracts were able to sensitize the resistant cells as well, which were derived from the parental HOS cells. Interestingly, the exposure of independent extracts of BAM-SX and AIM-SO showed enhanced cytotoxicity in the resistant cells, while a combination of both failed to impart significant effect. We assume that the combination has some mutually inhibitory effect in terms of their cytotoxic effect. We, thereafter, explored the mode of cell death in the parental cells and observed that they induce caspase-mediated apoptosis initiated primarily by alteration of intracellular redox levels. Interestingly, a prior inhibition of autophagy, enhanced cell death, suggesting a protective role of autophagy. The role of autophagy in cancer is controversially discussed, where it can act both as a tumor promoter or a stimulator of apoptotic cell death (27). Of late, a good number of studies have highlighted the role of autophagy in survival of cancer cells under cellular stress (27, 28). We analyzed the conventionally studied autophagic marker-LC3-BII and observed an accumulation of the same, indicative of enhanced autophagy upon exposure to extract; and an increase in autophagy served as a survival strategy adopted by the cells under the current scenario. Furthermore, HPTLC analysis resulted in identification of various phytochemicals such as berberine, palmitine, azadirachtin, nimbin, and nimbolide, which have been reported for anticancer properties and these compounds might be responsible for the observed activity of BAM-SX and AIM-SO. Our results suggest a high therapeutic potential of BAM-SX and AIM-SO against both sensitive and drug refractory HOS cancer cells and provides a mechanistic insight into their mode of action.

## Author Contributions

PS, SR, RC, and SM performed the experiments. RC, SM, and AP designed the study plan and wrote the manuscript. KL and HS performed the revision experiments.

## Conflict of Interest Statement

The authors declare that the research was conducted in the absence of any commercial or financial relationships that could be construed as a potential conflict of interest.

## References

[1] Demain AL Vaishnav P Natural products for cancer chemotherapy. Microb Biotechnol (2010) 4(6):687–99. 10.1111/j.1751-7915.2010.00221.x 21375717 PMC3815406

[2] Desai AG Qazi GN Ganju RK El-Tamer M Singh J Saxena AK Medicinal plants and cancer chemoprevention. Curr Drug Metab (2008) 9(7):581–91. 10.2174/138920008785821657 18781909 PMC4160808

[3] Siegel RL Miller KD Jemal A. Cancer statistics, 2016. CA Cancer J Clin (2016) 66(1):7–30. 10.3322/caac.21332 26742998

[4] Holohan C Van Schaeybroeck S Longley DB Johnston PG Cancer drug resistance: an evolving paradigm. Nat Rev Cancer (2013) 13(10):714–26. 10.1038/nrc3599 24060863

[5] Kubista B Schoefl T Mayr L van Schoonhoven S Heffeter P Windhager R Distinct activity of the bone-targeted gallium compound KP46 against osteosarcoma cells – synergism with autophagy inhibition. J Exp Clin Cancer Res (2017) 36:52–63. 10.1186/s13046-017-0527-z 28403890 PMC5389188

[6] Parsuram S Thing GS Dhanaraj SA. Polyherbal formulation: concept of ayurveda. Pharmacogn Rev (2014) 8(16):73–80. 10.4103/0973-7847.134229 25125878 PMC4127824

[7] Alzohairy MA. Therapeutics role of *Azadirachta indica* (Neem) and their active constituents in diseases prevention and treatment. Evid Based Complement Alternat Med (2016) 2016:11. 10.1155/2016/7382506 27034694 PMC4791507

[8] Paul R Prasad M Sah NK Anticancer biology of *Azadirachta indica* L (neem). Cancer Biol Ther (2013) 12(6):467–76. 10.4161/cbt.12.6.16850 21743298

[9] Potdar D Hirwani RR Dhulap S. Phyto-chemical and pharmacological applications of *Berberis aristata*. Fitoterapia (2012) 83(5):817–30. 10.1016/j.fitote.2012.04.012 22808523

[10] Azwanida NN A review on the extraction methods use in medicinal plants, principle, strength and limitation. Med Aromat Plants (2015) 4(3):1–6. 10.4172/2167-0412.1000196

[11] Złotek U Mikulska S Nagajek M Świeca M. The effect of different solvents and number of extraction steps on the polyphenol content and antioxidant capacity of basil leaves (*Ocimum basilicum* L.) extracts. Saudi J Biol Sci (2016) 23(5):628–33. 10.1016/j.sjbs.2015.08.002 27579013 PMC4992113

[12] Chowdhury R Chowdhury S Roychoudhury P Mandal C Chaudhuri K. Arsenic induced apoptosis in malignant melanoma cells is enhanced by menadione through ROS generation, p38 signaling and p53 activation. Apoptosis (2009) 14(1):108–23. 10.1007/s10495-008-0284-8 19082730

[13] Palvai S Nagraj J Mapara N Chowdhury R Basu S Dual drug loaded vitamin D3 nanoparticle to target drug resistance in cancer. RSC Adv (2014) 4(100):57271–81. 10.1039/C4RA06475E

[14] Mizushima N. Methods for monitoring autophagy. Int J Biochem Cell Biol (2004) 36(12):2491–502. 10.1016/j.biocel.2004.02.005 15325587

[15] Munafo DB Colombo MI. A novel assay to study autophagy: regulation of autophagosome vacuole size by amino acid deprivation. J Cell Sci (2001) 114(Pt 20):3619–29. 10.1242/jcs.156034 11707514

[16] Patel MC Rathod IS Sindhu BE HPTLC method for estimation of berberine in ayurvedic formulations containing *Berberis aristata* by an acid dye method. Int J Pharm Pharm Sci (2013) 5(1):129–31.

[17] Mohammadreza S Zdenek S Gianlucca M Lourdes BC Leopod LI. Matrix-free thin-layer chromatography/laser desorption ionization mass spectrometry for facile separation and identification of medicinal alkaloids. Rapid Commun Mass Spectrom (2009) 29:3655–60. 10.1002/rcm.4297 19899183

[18] Agarwal H Kaul N Paradkar AR Mahadik KR Standardization of crude extract of neem seed kernels (*Azadirachta indica* A. Juss) and commercial neem based formulations using HPTLC and extended length PackedColumns SFC method. Chromatographia (2005) 62:183–95. 10.1365/s10337-005-0588-6

[19] Mishra SK Raut KK Development of a sensitive HPTLC method for quantification of nimbolide in *Azadirachta indica* and its dosage form. J Chromatogr Sci (2013) 52(9):1–6. 10.1093/chromsci/bmt151 24108814

[20] Soni H Mishra K Sharma S Singhal AK Characterization of Azadirachtin from ethanolic extract of leaves of *Azadirachta indica*. J Pharm Res (2012) 5(1):199–201.

[21] Liou GY Storz P Reactive oxygen species in cancer. Free Radic Res (2010) 44(5):479–96. 10.3109/10715761003667554 20370557 PMC3880197

[22] Okon IS Zou MH. Mitochondrial ROS and cancer drug resistance: implications for therapy. Pharmacol Res (2015) 100:170–4. 10.1016/j.phrs.2015.06.013 26276086 PMC4893310

[23] Liu JF Hou CH Lin FL Tsao YT Hou SM. Nimbolide induces ROS-regulated apoptosis and inhibits cell migration in osteosarcoma. Int J Mol Sci (2015) 16(10):23405–24. 10.3390/ijms161023405 26426012 PMC4632706

[24] Mantena SK Sharma SD Katiyar SK. Berberine, a natural product, induces G1-phase cell cycle arrest and caspase-3-dependent apoptosis in human prostate carcinoma cells. Mol Cancer Ther (2006) 5(2):296–308. 10.1158/1535-7163.MCT-05-0448 16505103

[25] Hao F Kumar S Yadav N Chandra D. Neem components as potential agents for cancer prevention and treatment. Biochim Biophys Acta (2014) 1846(1):247–57. 10.1016/j.bbcan.2014.07.002 25016141 PMC4734358

[26] Nishino H Kitagawa K Fujiki H Iwashima A. Berberine sulfate inhibits tumor-promoting activity of teleocidin in two-stage carcinogenesis on mouse skin. Oncology (1986) 43(2):131–4. 10.1159/000226349 3081844

[27] Sui X Chen R Wang Z Huang Z Kong N Zhang M Autophagy and chemotherapy resistance: a promising therapeutic target for cancer treatment. Cell Death Dis (2013) 4:e838. 10.1038/cddis.2013.350 24113172 PMC3824660

[28] Yang ZJ Chee CE Huang S Sinicrope FA. The role of autophagy in cancer: therapeutic implications. Mol Cancer Ther (2011) 10(9):1533–41. 10.1158/1535-7163.MCT-11-0047 21878654 PMC3170456

[29] Wang N Feng Y Zhu M Tsang CM Man K Tong Y Berberine induces autophagic cell death and mitochondrial apoptosis in liver cancer cells: the cellular mechanism. J Cell Biochem (2010) 111(6):1426–36. 10.1002/jcb.22869 20830746

[30] Wang J Qi Q Feng Z Zhang X Huang B Chen A Berberine induces autophagy in glioblastoma by targeting the AMPK/mTOR/ULK1-pathway. Oncotarget (2016) 7(41):66944–58. 10.18632/oncotarget.11396 27557493 PMC5341849

[31] Subramani R Gonzalez E Arumugam A Nandy S Gonzalez V Medel J Nimbolide inhibits pancreatic cancer growth and metastasis through ROS-mediated apoptosis and inhibition of epithelial-to-mesenchymal transition. Sci Rep (2015) 6:19819 10.1038/srep19819 PMC4726267 26804739

[32] Foucquier J Guiedj M. Analysis of drug combinations: current methodological landscape. Pharmacol Res Perspect (2015) 3(3):1–11. 10.1002/prp2.149 26171228 PMC4492765

[33] Bielack SS Carrle D Hardes J Schuck A Paulussen M. Bone tumors in adolescents and young adults. Curr Treat Options Oncol (2008) 9(1):67–80. 10.1007/s11864-008-0057-1 18449804

[34] Hughes DP. Strategies for the targeted delivery of therapeutics for osteosarcoma. Expert Opin Drug Deliv (2009) 6(12):1311–21. 10.1517/17425240903280422 19761419 PMC4163784

[35] Sun Y Xun K Wang Y Chenc X A systematic review of the anticancer properties of berberine, a natural product from Chinese herbs. Anticancer Drugs (2009) 20:757–69. 10.1097/CAD.0b013e328330d95b 19704371

[36] Wu J Xiao Q Zhang N Xue C Leung AW Zhang H Palmatine hydrochloride mediated photodynamic inactivation of breast cancer MCF-7 cells: effectiveness and mechanism of action. Photodiagnosis Photodyn Ther (2016) 15:133–8. 10.1016/j.pdpdt.2016.07.006 27444887

[37] Sanders M Liu A Li T-K Wu H-Y Desai S Mao Y Selective cytotoxicity of topoisomerase-directed protoberberines against glioblastoma cells. Biochem Pharmacol (1998) 56(9):1157–66. 10.1016/S0006-2952(98)00243-3 9802326

[38] Bodduluru LN Thota NJ Borua CC Sistala R Chemopreventive and therapeutic effects of nimbolide in cancer: the underlying mechanisms. Toxicol In Vitro (2014) 4(11):1–6. 10.1016/j.tiv.2014.04.011 24759803

[39] Nielsen CA Emdin SO Landberg G Cycin E overexpression, a negative prognostic factor in breast cancer with strong correlation to oestrogen receptor status. Br J Cancer (1996) 74:874–80. 10.1038/bjc.1996.451 8826852 PMC2074748

